# Dynamic behavior of rearranging carbocations – implications for terpene biosynthesis

**DOI:** 10.3762/bjoc.12.41

**Published:** 2016-02-29

**Authors:** Stephanie R Hare, Dean J Tantillo

**Affiliations:** 1 Department of Chemistry, University of California–Davis, 1 Shields Avenue, Davis, CA 95616, USA

**Keywords:** carbocation, density functional theory, dynamics, mechanism, terpene

## Abstract

This review describes unexpected dynamical behaviors of rearranging carbocations and the modern computational methods used to elucidate these aspects of reaction mechanisms. Unique potential energy surface topologies associated with these rearrangements have been discovered in recent years that are not only of fundamental interest, but also provide insight into the way Nature manipulates chemical space to accomplish specific chemical transformations. Cautions for analyzing both experimental and theoretical data on carbocation rearrangements are included throughout.

## Review

### Introduction to terpene forming carbocation rearrangements

**Figure 1:**
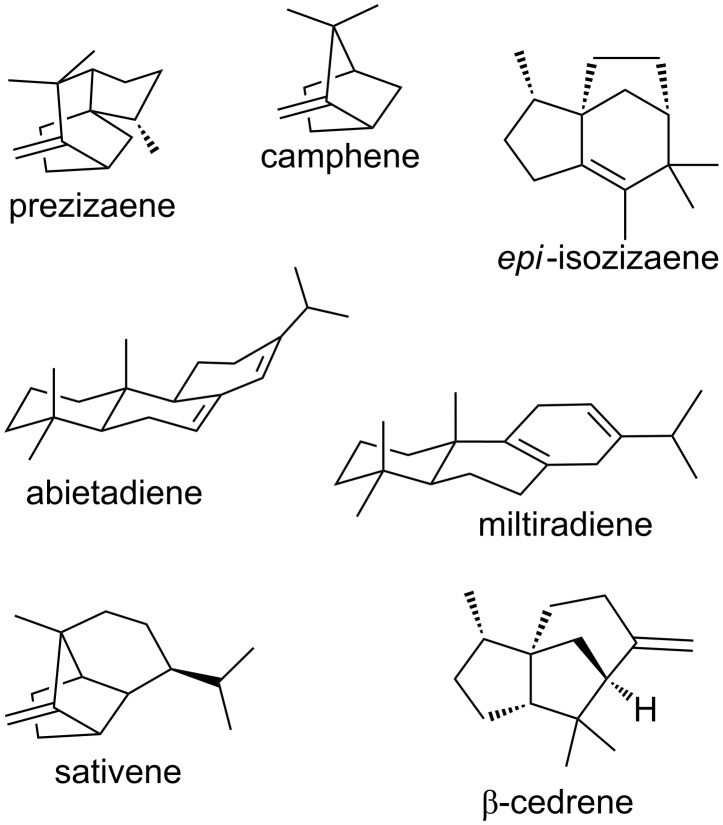
Representative terpenes.

Terpene natural products display a striking range of molecular architectures, varying in size and complexity (Figure 1) [1–5]. Some terpenes sport multiple stereogenic centers and multiple carbocyclic rings. These complex hydrocarbon frameworks are derived, however, from simple precursors lacking stereogenic centers and rings that are transformed in only one or two enzyme-promoted reactions. These reactions involve generation of a carbocation by protonation or loss of a diphosphate group followed by cyclization, alkyl shift, hydride shift and/or proton transfer reactions to generate new, more complex, carbocations. Ultimately these carbocations are either trapped by a nucleophile (e.g., water, diphosphate) or deprotonated to form alkenes.

The details of terpene-forming carbocation cyclization/rearrangement processes have been of interest for decades [1–6]. Although much has been learned, new observations continue to surprise researchers in the natural products field. For instance, recent computational/theoretical studies have focused on the inherent dynamical behavior of carbocations involved in these reactions – the subject of this review article. These studies have revealed that inherent dynamical tendencies, i.e., the dynamical behavior of carbocations in the absence of an enzyme, tend to be reflected in product distributions for enzyme-promoted reactions. Consequently, the problem of elucidating the role of terpene synthase enzymes in terpene formation has been redefined. In addition, these studies have pointed to the possibility that inherent dynamical tendencies of reactive intermediates may play important roles in enzyme evolution.

Here we review key studies on the dynamical behavior of carbocations. First we provide an introduction to dynamical behavior and how it is examined using modern theoretical tools. Then we describe studies dealing with carbocations that are not involved in terpene formation, but which reveal reactivity principles that may have implications for terpene biosynthesis. This is followed by descriptions of the relatively few studies published so far that are concerned with dynamical behavior of carbocations involved in terpene-forming reactions. In each section, we highlight important take home messages.

### Dynamical behavior – a brief tutorial

**Figure 2:**
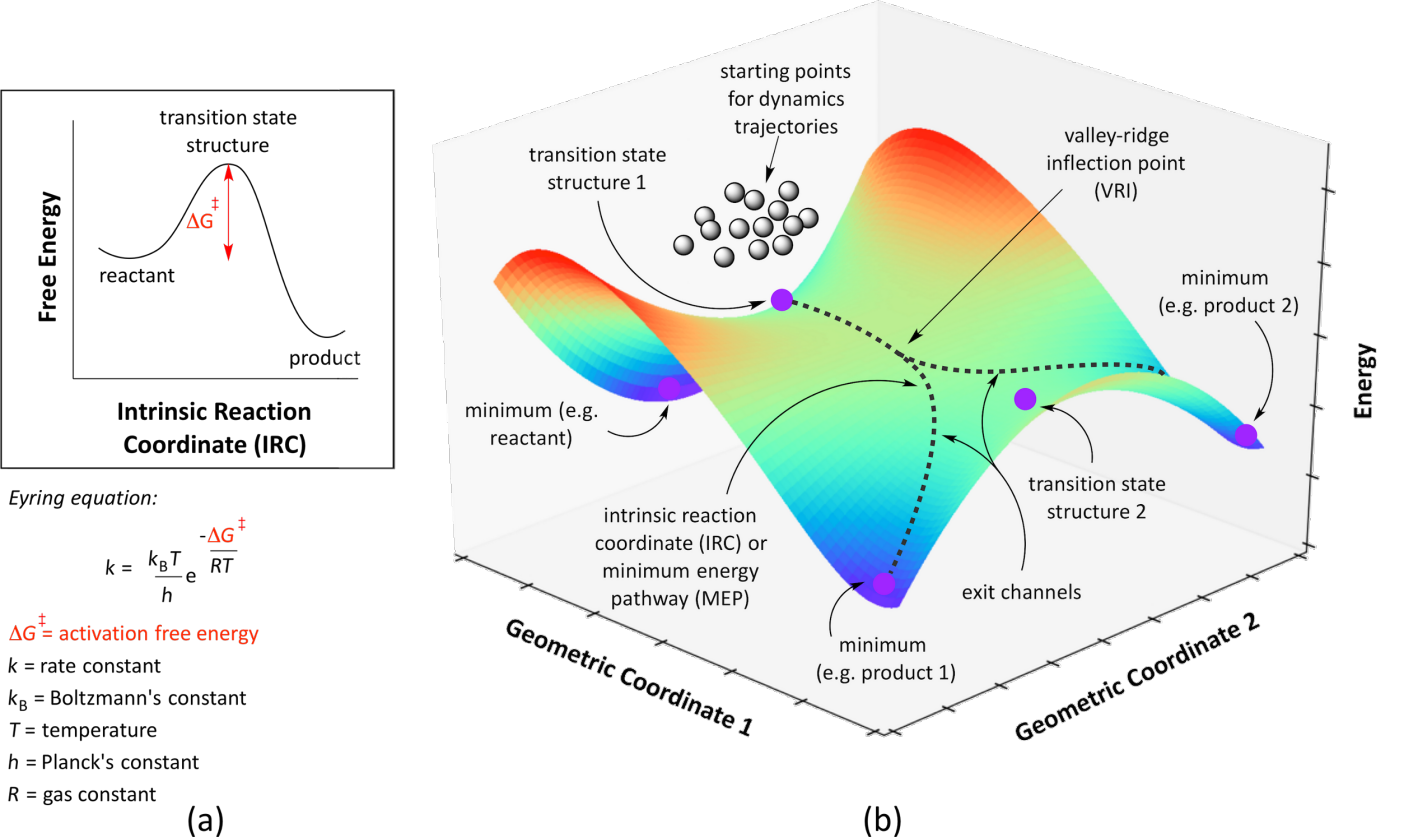
Two different models showing how energy evolves throughout the course of a reaction: (a) a two-dimensional plot, where the reactant follows a single path through the TSS to the product at a rate governed by the Eyring equation [9] and (b) a three-dimensional hypothetical PES exhibiting the features of a PTSB and a qualitative representation of the starting points for dynamics trajectories. The function z = 2x^5^ − 5x^2^ − 5xy + y^2^ + 2 was used to generate this hypothetical surface.

The reactivity of a molecule often ties back to a single characteristic: its energy (in particular, its free energy). Computational and synthetic chemists are most often interested in potential energy because selective conversion of the potential energy associated with chemical bonds is the basis of chemical reaction design. The surface representing how the potential energy of a molecule is affected by geometrical (and subsequently electronic) changes is called (unsurprisingly) the molecule’s potential energy surface (PES). Technically, there are 3*N* dimensions in which geometrical changes can occur, where *N* is the number of atoms in the molecule each moving in 3 dimensions. When all *N* atoms move in the x, y, or z directions, the molecule is translating. Similarly, if all *N* atoms are rotating along the x, y, or z axes, the entire molecule is rotating. This leaves 3*N* − 6, or 3*N* − 5 if a molecule is linear, vibrational degrees of freedom that contribute to the molecule’s internal energy. Being able to visualize how each of these changes affects the energy of the molecule would require the ability to visualize (3*N* − 6) + 1-dimensional space. However, (3*N* − 6) + 1 dimensions can be reduced to two dimensions by looking only at the minimum energy pathway (MEP) between two minima on the PES, which is also referred to as the intrinsic reaction coordinate (IRC; Figure 2, left) [7–8]. It is the IRC that is typically used to make arguments for reactivity observed experimentally.

The IRC contains a wealth of information about the behavior of a particular system, but not all chemical phenomena can be explained by analyzing this pathway alone. The most common characteristic of an IRC that is used to make arguments for relative reaction rates leading to chemo-, regio-, or stereoselectivity of a reaction is the energy difference between the reactant and the relevant transition state structure (TSS) along the IRC. Traditional static approaches, transition state theories (TSTs) [9–13] and the Rice–Ramsperger–Kassel–Marcus theory (RRKM) [14–17], that relate activation barriers to reaction rates rely on the assumption that the molecule will follow the IRC at all times during a chemical reaction (sometimes referred to as “quasi-equilibrium conditions”). Importantly, this pathway lies on the PES and thus neglects the kinetic energy of the system. Kinetic energy becomes particularly important when the PES topology exhibits certain features that can make the system deviate from the IRC, such as: (1) when a reaction pathway involves a shallow intermediate (particularly when the preceding TSS is high in energy) and (2) when a single TSS leads directly to multiple minima, sometimes called an “ambimodal” TSS [18], without intervening minima; this scenario is referred to as a pathway with one or more post-transition state bifurcations (PTSB) [19–26]. For a detailed discussion of unique PES features that lead to deviations from IRC behavior, see Birney’s review on PESs of pericyclic and pseudopericyclic reactions [27].

**Figure 3:**
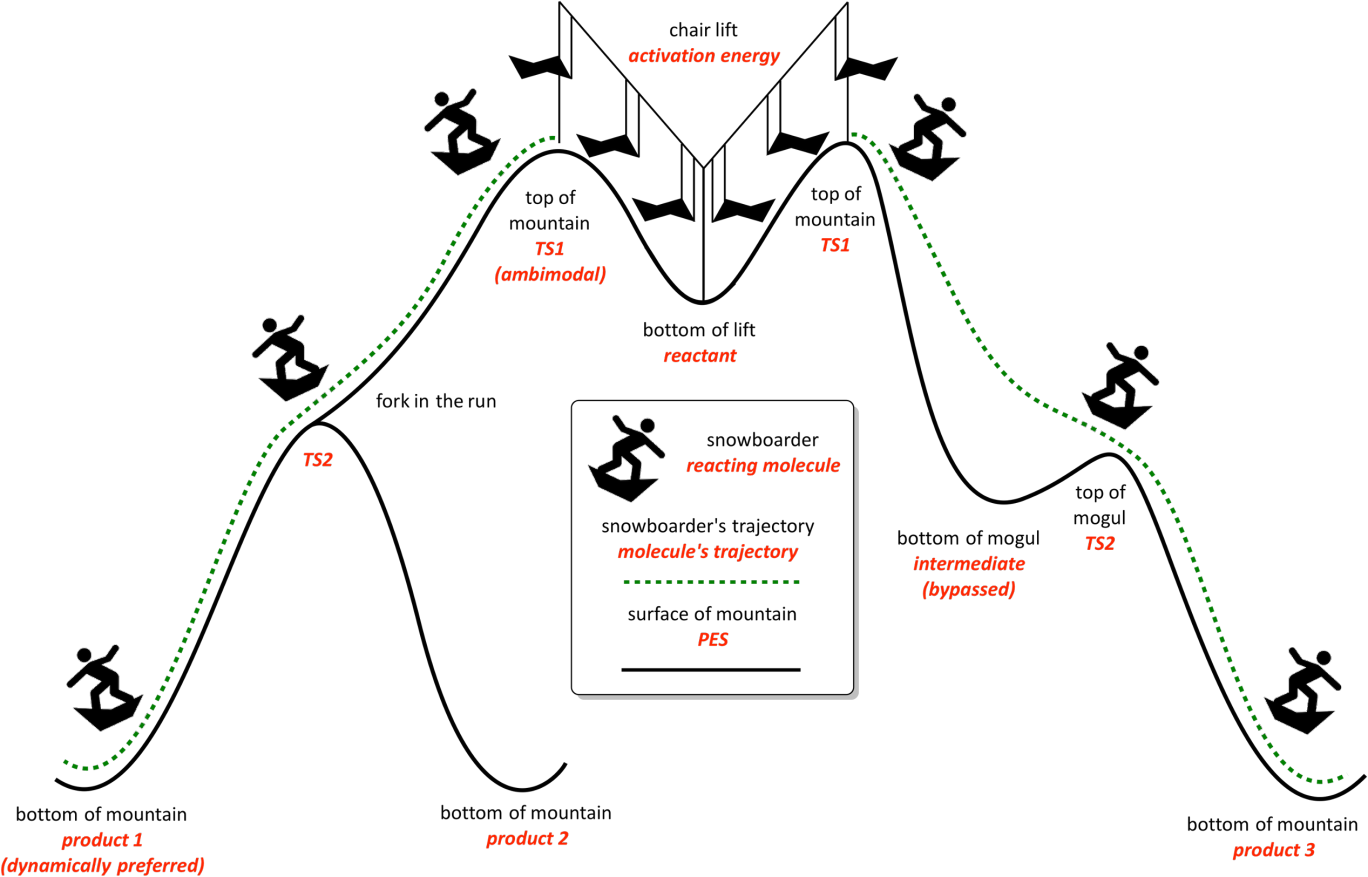
A depiction of the “snowboarder” analogy for reactions displaying non-statistical dynamic effects. Features of a PES that correspond to features found on the slopes are highlighted in red. This figure illustrates two independent phenomena: Left: formation of a preferred product following an ambimodal TSS due to dynamic matching. Right: an intermediate that is rapidly passed through or bypassed as a result of dynamic matching.

These two scenarios are visualized by way of an analogy in Figure 3. First, consider scenario (1). Imagine a snowboarder riding down a mountain. If the mountain is very tall and there is a mogul on the way to the bottom (Figure 3, right), the snowboarder is more able to easily pass the small hill than if he or she started from the base of the mogul. At the molecular level, this scenario can result in bypassed intermediates, i.e., an IRC having a minimum calculated along the pathway to the product, but with a lifetime that is not long enough to allow for equilibration; some pathways/trajectories will also skirt past the deepest parts of the energy well. Additionally, if the initial path down the mountain splits into two paths to the bottom of the mountain (i.e., at the molecular level, having an ambimodal TSS; Figure 3, left), it will be easier for the snowboarder to take the path that requires fewer changes in direction, unless he or she is leaning heavily toward the other path. In both scenarios, where the snowboarder (molecule) came from and how it was behaving (vibrating) on its way to the shallow valley (minimum) or fork in the path (PTSB) influences the path ultimately taken and the time associated with doing so. This concept, at the molecular level, is referred to as “dynamic matching” [28]. Molecules similarly retain momentum within particular vibrational modes if the timescale of the reaction is too short for the molecule’s kinetic energy to be distributed statistically throughout all vibrational modes. Reactions that undergo generation of reactive intermediates often meet this criterion and exhibit what are called “non-statistical dynamic effects”, that is, product distributions that cannot be rationalized by traditional TST [19,29–30]. These effects (highlighted through the examples discussed below) are typically described using classical mechanics (i.e., solving either Newton’s or Hamilton’s classical equations of motion to propagate nuclear positions), but there have been cases reported where *quantum* dynamic effects have been found to be important, particularly when tunneling effects contribute significantly to the reaction rate [31–34].

To acquire evidence for non-statistical dynamic effects, molecular dynamics (MD) simulations are run for a statistically relevant number of trajectories (typically on the order of hundreds or thousands, depending on the system and the starting point for trajectories) [35–36]. The most common modern technique for computing dynamics trajectories for organic reactions is the method of direct dynamics. With direct dynamics, instead of solving for a PES analytically, each point along a trajectory is calculated numerically “as needed” or “on-the-fly”. A quantum chemical program capable of ab initio or density functional theory (DFT) calculations is used to calculate either (1) force constants (via frequency calculation) along the trajectory, either at every point or in periodic increments, or (2) the gradient of the potential energy, depending on the specific integrator chosen to integrate the equations of motion. The calculation of gradients rather than force constants is significantly faster, but requires a smaller time step to achieve the same calculation accuracy. The calculations are run under the Born–Oppenheimer Approximation, which is why they are also called Born–Oppenheimer Molecular Dynamics (BOMD) calculations, so that nuclear motion and electronic structure are calculated separately, the former propagated classically and the latter determined using quantum mechanics.

As with any computational (or experimental) study, there will always be a tradeoff between sampling a sufficient amount of the relevant chemical space and completing the study in a reasonable amount of time. Different strategies can be used to achieve a compromise between these factors, depending on the size of the system of interest and the accuracy required to answer the relevant chemical questions. MD simulations have been employed to answer two different questions about the chemical reactions discussed below: (1) what mechanism(s) is energetically viable? and (2) do (non-statistical) dynamic effects exert control over product distributions? While trajectories can be started from anywhere on a PES, it is most common to initiate trajectories either from a structure that is a minimum (usually the reactant for the reaction of interest) – used when exploring possible mechanisms – or a TSS – used when assessing the impact of dynamic effects for a particular mechanism. In both cases, each atom in the molecule is given a random initial velocity and each vibrational mode is displaced a random distance, such that the total kinetic and potential energy of the molecule is equal to the amount of energy available at the specified temperature. The problem with initiating trajectories from a minimum, however, is that there is no guarantee the trajectories are going to be “productive”. This creates an operational problem in most cases because, relative to the optimization of stationary points on a PES, MD trajectories are very computationally expensive, a result of having to repeatedly calculate force constants. For a 1 ps long direct dynamics trajectory with a time step of 1 fs where force constants are calculated at each point, the nuclear and electronic structure of the molecule will need to be recalculated a total of 1000 times, which equates to a great deal of computer time, even in 2016. There is a (somewhat controversial) method to facilitate barrier crossing in which a “biased potential” is employed to “push” a reactant up and toward the barrier of interest in an MD simulation [37–39]. The controversy arises from the question of whether such a biased method leads to biased results, so using a biased method requires testing against unbiased methods and/or experimental data to ensure accuracy. The complication of having unproductive trajectories is mitigated when initiating trajectories from a transition state, but of course this leads to the most biased strategy of all because a pre-determined TSS is the starting point for such a calculation. This strategy cannot be used to explore a large variety of possible mechanisms, but is effective for determining the magnitude of dynamic effects associated with falling downhill from a particular TSS. Therefore, one can make the assumption that the system always passes through the transition state region when only “reactive” trajectories are of interest. Notably, this makes the assumption that quasi-equilibrium conditions are followed up until the transition state region. For most systems, this is a reasonable assumption, but careful consideration of any chemical steps in the reaction preceding the transition state from which trajectories are initiated should be made, since any dynamical effects preceding the transition state would be neglected and would have to be treated separately if of interest. Studies involving trajectories initiated from minima and transition states have both been carried out on carbocations [25] and examples of each are described below. While many different quantum chemical methods can be used to carry out trajectory calculations, standard density functional theory (DFT) approaches are most commonly used [35–40]. In particular, the B3LYP and mPW1PW91 functionals, along with small to medium sized basis sets have seen the most use in studying carbocation rearrangements of relevance to biosynthesis [6].

**Figure 4:**
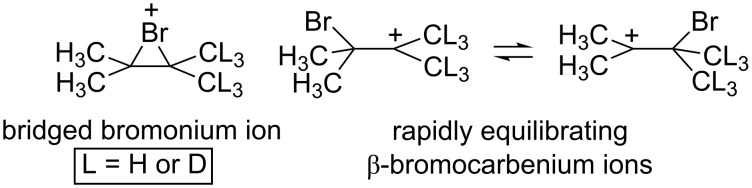
The tetramethylbromonium ion system [14].

Using molecular dynamics trajectories to rationalize experimental results is still not standard practice, but the potential for the utility of dynamics simulations in a variety of systems has certainly been demonstrated. The studies detailed below primarily highlight situations where molecular dynamics simulations were used to quantify “non-IRC” behavior, but the value of dynamics simulations does not stop there. For example, Bogle and Singleton used dynamics trajectories to gather evidence for whether the tetramethylbromonium ion existed as a single *C*_2_*_v_*-symmetric bridged structure or rapidly interconverted between two β-bromocarbenium ion structures (Figure 4) [41]. Experimental evidence for which of these two types of scenarios is present is generally obtained using the “isotopic perturbation” method pioneered by Saunders [42–44]. In this method, isotopic labels are added (e.g., L = D in Figure 4) and NMR spectra are acquired. The ^13^C NMR spectrum of the resultant system would be expected to exhibit a large difference in signals (Δ) between carbons with H versus D substituents, whereas essentially no difference in signals between carbons would be expected if there was no equilibrium to affect. Ohta et al. [45] experimentally determined a large Δ (3.61 ppm) for the system shown in Figure 4, concluding that the two β-bromocarbenium ion structures interconvert in solution. However, by running dynamics simulations on the system and calculating NMR chemical shifts at each point, Bogle and Singleton were able to gather evidence that this effect instead can be attributed to geometrical changes of a bridged ion resulting from the isotopic substitution. They concluded that it cannot be assumed that a large Δ resulting from isotopic labeling guarantees rapid equilibration between two unlabeled structures. While the cases described below are focused on reaction pathways, similar cautions on interpretation are presented throughout. We hope these cautions will encourage a healthy skepticism in the interpretation of all data, experimental and computational alike.

***Take home messages:***

• A PES can reveal important information about a system, but complicating features on some PESs make analyses using traditional TST incomplete.

• Two common examples of these complicating features are (1) highly exergonic steps leading to bypassed intermediates and (2) PTSBs.

• Molecular dynamics simulations can be used in these contexts to provide evidence for the pathways that are accessible to the molecular system given a particular amount of initial kinetic energy. These simulations can be initiated from the region of the reactant or TSS, but which is appropriate for a specific case depends on the nature of the chemical questions to be answered.

### Non-biological carbocation rearrangements

#### Generation of carbocations via protonated alcohols – the concerted vs stepwise spectrum

**Figure 5:**
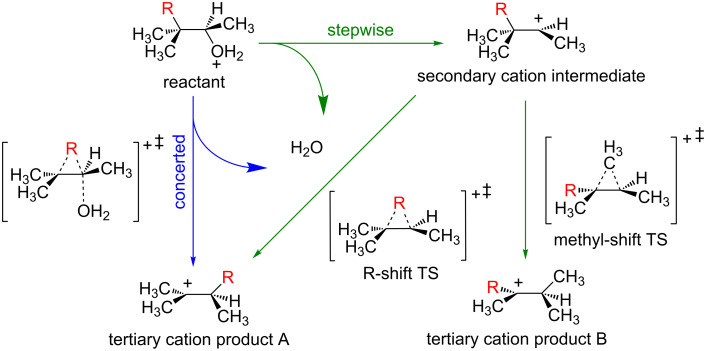
The reaction mechanisms of interest in the PES and dynamics studies of Dupuis and co-workers (R = CH_3_) and de Souza et al. (R = CH_3_, Et, iPr). Note: in the case of R = CH_3_, tertiary cation products A and B are equivalent. Adapted from Dupuis and co-workers and de Souza et al. [46,55].

The seminal work of Dupuis and co-workers in running dynamics simulations to elucidate the nature of the dehydration-rearrangement mechanism of protonated pinacolyl alcohol (Figure 5, R = CH_3_) was instrumental in bringing the issue of dynamic effects to a wide audience [46]. The question addressed in this work was ostensibly simple: is the mechanism of dehydration/alkyl migration of a protonated alcohol a concerted or stepwise process? The IRC for the process revealed a concerted mechanism (Figure 5, blue), with no secondary carbocation found as a stationary point on the PES. However, molecular dynamics simulations initiated from the reactant revealed trajectories that predominantly followed a stepwise mechanism (Figure 5, green), with a lifetime of the secondary carbocation of up to 4000 fs. This is the opposite of the situation illustrated on the right side of Figure 3; instead of an intermediate structure being rapidly bypassed due to dynamic effects, the reacting molecule gets stuck in a region of the PES where there is no minimum. In total, 50 trajectories were run where, after 500 fs, 20 trajectories went to the secondary carbocation, only one trajectory went directly to the rearranged product (concerted mechanism), and one remained in the secondary carbocation region before eventually affording the rearranged product. The remaining 28 trajectories remained in the reactant region, illustrating the complication associated with initiating dynamics trajectories from a minimum on the PES mentioned above. Though this number of productive trajectories would not be considered sufficient to make definitive conclusions regarding the experimental behavior of this system (especially given the computational power available today), this study paved the way for future dynamics studies and correctly predicted that “similar findings will arise for many other reactions … and interpretation of reaction mechanisms ought to consider the effects of dynamics explicitly” [46]. In light of more recent studies (e.g., see below), the results just described could be anticipated. The IRC for the dehydration-rearrangement reaction actually proceeds through the region where the secondary carbocation resides, even though this structure is not a PES minimum. The curvature of the IRC in this region would likely have indicated the presence of a “hidden intermediate” [47–51], i.e., a structure along the IRC that is not a minimum but is associated with an energy plateau and may have a substantial lifetime. Such IRCs have subsequently been observed for many reactions for which secondary carbocations are putative intermediates [52–54].

More recently, de Souza et al. revisited these systems and conducted a study looking at the rearrangement behavior of a series of protonated alcohols using TST, a “static” approach, and a slightly different variation of molecular dynamics simulations compared to that used by Dupuis and co-workers [55]. Additionally, replacing R in Figure 5 with a non-methyl substituent opened up the possibility of the formation of two different products resulting from migration of different alkyl groups (tertiary carbocation products A and B in Figure 5). While these differences led to results that were quantitatively different from those described in the Dupuis study, they were qualitatively the same and led the authors to essentially the same conclusions. The authors emphasized that, in reality, all mechanisms are on a spectrum, where “concerted” and “stepwise” define limiting cases, in line with previous descriptions of carbocation reactions as existing on a “continuum” [56–57]. In the case of the dehydration-rearrangements of protonated alcohols, the most intense “band” in the spectrum of possible reaction types involves the formation of a secondary carbocation structure prior to formation of the rearranged product, as revealed by molecular dynamics simulations.

***Take home messages:***

*• Dynamics simulations can reveal behavior not readily apparent in IRC calculations *[58]*.*

• The terms “concerted” and “stepwise” define the limiting cases of a spectrum/continuum of mechanistic possibilities.

#### Norborn-2-en-7-ylmethyl cation – memory effects

**Figure 6:**
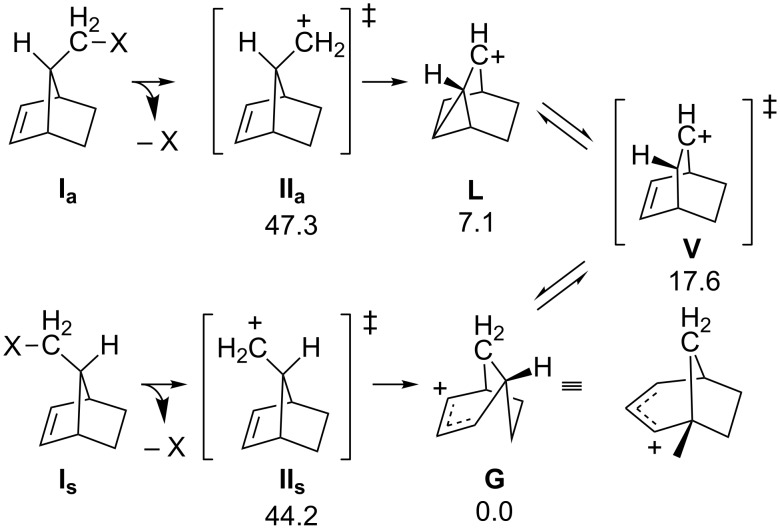
The portion of the norborn-2-en-7-ylmethyl cation PES examined by Ghigo et al. [60]. Energies reported are electronic energies, including zero-point corrections (ZPE), at the B3LYP/6-31G(d) level of theory and are all relative to that of **G** [61–63].

Dynamic effects are often suspected when a stereochemical result is observed experimentally that is inconsistent with a proposed mechanism, despite other evidence supporting the proposed mechanism. For example, Berson et al. discovered that solvolysis of *syn-* and *anti-*norborn-2-en-7-ylmethyl-X diastereomers (**I_s_** and **I_a_**, Figure 6; X is a leaving group) both led to the same two products, but in different ratios, despite sharing a common intermediate (in different conformations; **V**, Figure 6) [59]. The major product generated from the solvolysis of **I_a_** was the acetate of carbocation **L**, with a small amount of the acetate of carbocation **G** also observed. Solvolysis of **I_s_** also led to the acetate of carbocation **L**, but this time accompanied by a significant amount of the acetate of carbocation **G**. This difference in product distribution (whose magnitude varied with leaving group identity) was ascribed to a “memory effect”. Put simply, product ratios were skewed from what would be expected by simply comparing activation barriers, because the reacting molecule “remembers” the conformation from which it came; this is a hallmark of dynamic matching. Additionally, the memory effect can be decreased by “leakage” when one conformation of the common intermediate rapidly converts to the other conformation (essentially the equilibration expected for a reaction not displaying non-statistical dynamic effects).

Ghigo et al. set out to explore the memory effect phenomenon computationally [60]. The relevant PES for this transformation (key points shown in Figure 6) was examined using several DFT methods. The portion of the PES prior to formation of TSSs **II_a_** and **II_s_** was also explored, but it was assumed that all structures were required to go through TSSs **II_a_** and **II_s_** in order to make the products; consequently, dynamics trajectories were initiated from the regions of these TSSs (using a lower level of theory so that 250 trajectories from each transition state could be obtained in a reasonable amount of time; the influence of the leaving group on dynamical behavior was not explored). The results from the dynamics simulations were in qualitative agreement with the experimental results: trajectories initiated from **II_s_** generated almost equal amounts of cations **G** and **L**, while trajectories initiated from **II_a_** go predominantly to cation **L**.

***Take home message:***

• “Memory effects” can result from dynamic matching.

#### 2-Norbornyl and other highly delocalized cations – a caution on complexity

**Figure 7:**
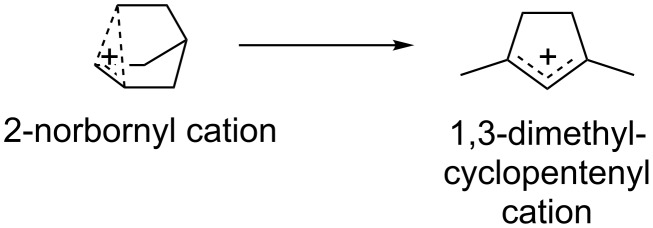
The transformation of 2-norbornyl cation to 1,3-dimethylcyclopentyl cation.

When exploring carbocation rearrangement mechanisms using MD simulations, one should remember that MD simulations are inherently statistical. That is, there are times when a systematic approach to exploring mechanistic pathways is preferable to MD simulations, which use random sampling techniques. This point is illustrated by two studies on the isomerization of the infamous 2-norbornyl cation to the 1,3-dimethylcyclopentenyl cation (DMCP^+^) (Figure 7) [64–65].

After an attempt by Mosley et al. to study the experimental IR spectrum of the 2-norbornyl cation in the gas phase revealed a structural rearrangement to DMCP^+^, Jalife et al. set out to determine the isomerization mechanism using modern computational methods [64]. BOMD simulations using DFT were employed, with trajectories initiated from the equilibrium geometry of the 2-norbornyl cation (i.e., the reactant structure) with random velocities assigned to all atoms. When a trajectory formed DMCP^+^, key points on the PES for the pathway observed in that trajectory were optimized. Two complex pathways to DMCP^+^ were found that had energy barriers that were reasonable given the experimental conditions used for generation of the 2-norbornyl cation. Both pathways involve a retro-Lawton–Bartlett “π-route” norbornyl ring-opening process [66–67]. The shorter mechanism was found to involve nine discrete chemical steps and had an overall predicted activation barrier of 33 kcal/mol, while the longer pathway involved 16 steps with an overall barrier of 37 kcal/mol. Similar results have been obtained for other complex carbocations: the same group used molecular dynamics to explore the homocubyl cation’s rearrangement behavior [68], and East et al. used “rising-temperature” molecular dynamics to determine the carbocation branching behavior of molecules relevant to petroleum chemistry [69–71].

Lobb also attempted to answer the same mechanistic question using a different strategy [65]. Instead of using BOMD simulations to explore possible pathways, Lobb wanted to “systematize” the mechanistic search to explore all possible isomerization pathways and predict their barriers. Lobb used simple algebraic tools, similar to a strategy employed by Johnson and others [72–76], to systematically generate a vast set of possible isomers of C_7_H_11_^+^ and rank them by their energies (calculated with DFT) [65,77]. The connectivity of each of the generated molecules was examined for isomorphism, ultimately leading to a set of 1254 distinct groups of isomers involved in possible rearrangements. This number is only an estimation of the full set of isomers, however, due to limitations of the automated methods. DMCP^+^ was found to be the global minimum for this set of isomers, consistent with experimental results [77]. The mechanistic pathways between isomers were explored by optimizing putative TSSs corresponding to breaking of each bond within a ring (if the molecule contains one) and hydride shifts. The 4500 unique TSSs optimized were then connected to the isomers they interconvert, connecting 1179 out of the 1254 carbocation isomers, to generate various pathways that led to the final product. A huge number of possible pathways were found, the shortest of which are summarized in Table 1.

**Table 1:** The number of pathways found by Lobb corresponding to a certain number of steps in the mechanism and the lowest overall activation barrier necessary for a pathway with that number of steps [65].

Number of steps	Number of paths	Lowest activation barrier (kcal/mol)

2	1	110.7
3	14	54.7
4	406	31.0
5	8460	29.3
6	171050	27.4

Though the MD strategy used by Jalife et al. uncovered two reasonable mechanistic pathways, the systematic approach taken by Lobb revealed 5 orders of magnitude more pathways that were shorter than those proposed by Jalife, many of which had a lower overall activation barrier, any number of which could be operative in the rearrangements of the 2-norbornyl cation to DMCP^+^. While a systematic search of all possible isomerization pathways should always be considered for carbocation rearrangements, it is often unnecessary (and prohibitively time-consuming) in the case of carbocation rearrangements that occur in Nature. Thankfully, enzyme-catalyzed carbocation rearrangements are often subject to conformational constraints that make analysis of the possible rearrangement pathways more tractable. Further discussion of enzymatic carbocation rearrangements is found below.

***Take home messages:***

• Sometimes there are many, many pathways that are energetically viable for the isomerization of a carbocation.

• In some cases, a systematic, rather than statistical, approach to determining all possible isomerization pathways is necessary to ensure that all energetically viable pathways have been explored.

• There is no “one-size-fits-all” strategy to "determine" a reaction mechanism using computations; however, coupled examination of PESs and simulations of dynamic effects can provide nearly (one hopes) exhaustive pictures of the transformation of reactants to products.

### Carbocation rearrangements that lead to terpenes

#### Camphene, sativene and prezizaene – lifetimes and electrostatic effects

**Figure 8:**
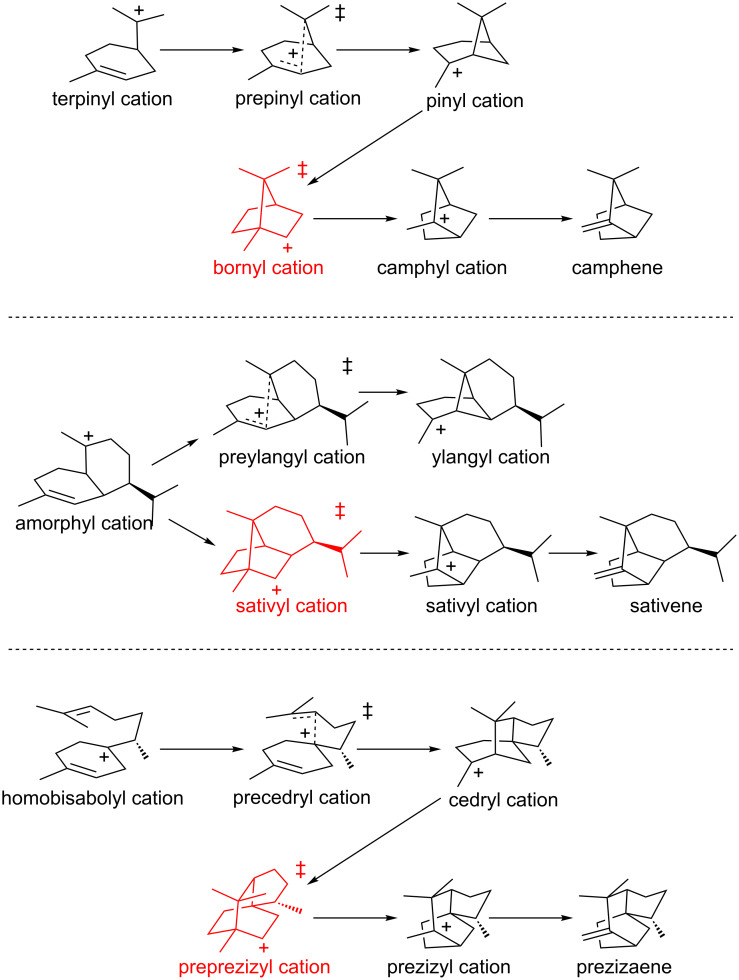
Carbocation rearrangements for which trajectory calculations were used to estimate lifetimes of secondary carbocations.

Portions of the C_10_H_17_^+^ and C_15_H_25_^+^ PESs (in the absence of enzyme) relevant to the formation of camphene [21–22,78], sativene [79] and prezizaene [54,80] (and related terpenes) were examined in detail using several DFT methods. For each of these systems, secondary carbocations were found along reaction coordinates, but they were not minima; rather, these structures resided in regions near to TSSs for concerted reactions involving the merging, asynchronously, of alkyl shift and/or cyclization events (Figure 8, red) [56–57]. Direct dynamics trajectory calculations were run on each of these systems, with trajectories initiated near the TSSs, i.e., the secondary carbocations. Trajectories (>100 for each system) were run in both forward and reverse directions. Based on the results of these calculations, average lifetimes for the secondary cations were found to range between 35 and 100 fs (with standard deviations between 10 and 35 fs), a time window on the same order as that for a single bond stretch. This lifetime could be increased significantly (by a factor of 2–3 for the bornyl cation, based on the preliminary calculations described) if the secondary carbocation engages in noncovalent interactions with electron rich groups (e.g., C–H···X hydrogen bonds [81]), thereby increasing the probability of trapping these species by deprotonation or addition of a nucleophile. Although some secondary carbocations have been found as minima in terpene-forming carbocation cyclization/rearrangement reactions [57], most are found near TSSs along reaction coordinates and therefore, as this study showed, can be expected to have exceedingly brief lifetimes in the absence of specifically oriented noncovalent interactions with groups in terpene synthase active sites. Molecular dynamics calculations using the full bornyl diphosphate synthase enzyme were also carried out (here using a combination of DFT and molecular mechanics) [21–22]. These simulations indicated that the bornyl cation also has a short lifetime in the active site of the enzyme, but one – 185 fs on average – that is longer (by approximately a factor of 4) than in the absence of the enzyme and complexed diphosphate.

***Take home messages:***

• Secondary carbocations, which often correspond to structures in the vicinity of transition states, tend to have short lifetimes, on the order of the period of a single-bond stretching vibration.

• These lifetimes can be increased via noncovalent interactions with electron-rich groups.

#### Abietadiene – navigating past forks in the road

**Figure 9:**
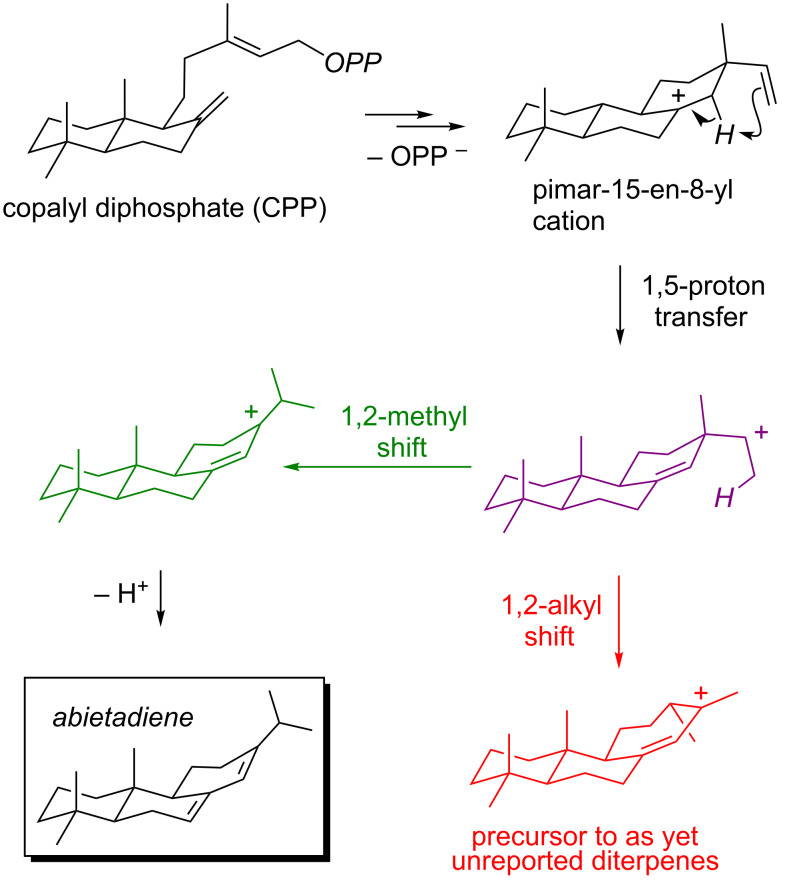
Carbocation rearrangements involved in abietadiene formation.

Pathways to abietadiene [82–90] have also been examined computationally [91–93]. First, the portion of the C_20_H_33_^+^ PES corresponding to the reactions depicted in Figure 9 was examined with several DFT methods [91]. This study revealed, quite unexpectedly, that intramolecular proton transfer in the pimar-15-en-8-yl cation can lead to a PTSB – one branch of which leads to the carbocation precursor to abietadiene (Figure 9, green), but the other branch of which leads to a rearranged skeleton, not yet reported for any diterpenes/diterpenoids from Nature (Figure 9, red). Interconversion of these two carbocations proceeds via a TSS that resembles the secondary carbocation expected to be formed upon proton translocation (Figure 9, purple), i.e., the secondary carbocation again corresponds to a TSS rather than a minimum. Direct dynamics trajectories were run from the 1,5-proton transfer transition state region, using both small model carbocations and full-sized structures and using several theoretical methods [92–93], and a ratio of trajectories leading to the abietadiene precursor versus the rearranged carbocation of 1.1–1.7:1 was found. These results first indicate that there is an inherent dynamical tendency built into the substrate (an enzyme was not present during the simulations) for formation of the observed natural product. Second, these results indicate that the inherent dynamical preference is not large enough to rationalize why abietadiene synthase produces 95% abietadiene (and simple diene isomers) [87], setting the stage for future studies aimed at elucidating the means by which abietadiene synthase steers its reaction away from rearrangement and at engineering abietadiene synthase so that it selectively forms rearranged products.

***Take home messages:***

• PTSBs can occur in biosynthetically-relevant carbocation rearrangements.

• There is an inherent dynamical tendency of the carbocations involved in abietadiene formation to form abietadiene, even in the absence of an enzyme.

• There is also an inherent dynamical tendency of the carbocations involved in abietadiene formation to form a rearranged product, that has not yet been observed in Nature, in a similar magnitude.

• Direct enzymatic intervention is likely necessary to overcome the latter tendency, although the nature of this intervention has not yet been characterized.

#### Miltiradiene – multiple sesquential bifurcations and testable predictions

**Figure 10:**
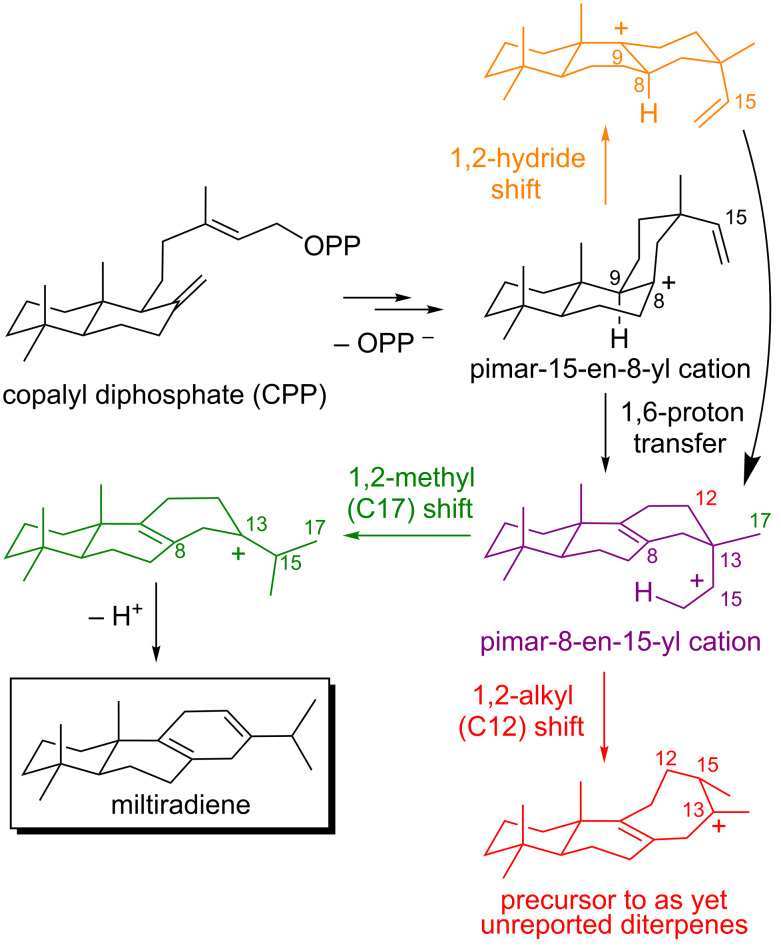
Carbocation rearrangements involved in miltiradiene formation.

The PES associated with formation of miltiradiene (Figure 10) [94], interrogated with a variety of DFT methods, was also found to involve a PTSB following a proton transfer TSS [26]. Surprisingly, however, this bifurcation was associated with a complex PES with flat regions and multiple additional sequential bifurcations. As a result, direct pathways from the 1,6-proton transfer TSS to eight products, without the intermediacy of any PES minima, were found. This is an unusual reactivity problem for an enzyme to tackle! How is one carbon skeleton obtained in high yield when barrierless pathways to eight different skeletons emanate from the same TSS? Direct dynamics trajectory calculations were again applied, with trajectories initiated in the region of the 1,6-proton transfer TSS (specifically for proton transfer to the *re* face of the C=C double bond) [95]. Although pathways to many products exist on the PES, only two products were formed to any appreciable extent in the dynamics calculations – the carbocation precursor to miltiradiene (Figure 10, green) and, similar to the scenario described above for abietadiene, a rearranged carbocation with a skeleton not yet reported in any natural products (Figure 10, red). These two carbocations were predicted to form in approximately a 1:1 ratio. Again, there is an inherent dynamical tendency for the substrate to form the observed natural product, but again this tendency is not strong enough to preclude formation of a rearranged product. In addition, the dynamics calculations indicated that the 1,2-methyl (C17) shift that forms the abietadiene precursor should occur specifically to one face of the carbocation carbon (C15), a prediction that could be tested through substrate labeling. If only trajectories that lead to the pimar-15-en-8-yl cation are considered, then a product ratio of approximately 2:1, in favor of miltiradiene formation, is found, i.e., some trajectories actually connect to a carbocation formed by a 1,2-hydride shift of the pimar-15-en-8-yl cation (Figure 10, orange). This result suggests that preorganization of the substrate into a conformation that disfavors the 1,2-hydride shift actually promotes miltiradiene formation. Finally, when dynamics trajectories were initiated from the region of the 1,6-proton transfer transition state associated with proton migration to the *si* face of the C=C double bond, the carbocation precursor to abietadiene was formed <1% of the time. This result implies that the pimar-15-en-8-yl cation is bound in a conformation that allows for proton transfer specifically to the *re* face of the C=C double bond. This study serves to redefine the problem faced by miltiradiene synthase in controlling selectivity, makes firm predictions about the bound conformation of the substrate and the stereochemical course of the enzymatic reaction from calculations that did not include the enzyme, and again sets the stage for future rational reengineering efforts.

***Take home messages:***

• Multiple sequential PTSBs can occur in biosynthetically-relevant carbocation rearrangements.

• The PES for miltiradiene formation (in the absence of an enzyme) involves direct pathways from a single TSS to many products.

• There is, however, an inherent dynamical tendency of the carbocations involved in miltiradiene formation (in the absence of an enzyme) to form almost exclusively miltiradiene and a rearranged product that has not yet been observed in Nature in comparable amounts.

• Direct enzymatic intervention is likely necessary to reduce the dynamical tendency to form the rearranged product. Although the nature of this intervention has not yet been deduced, it likely involves conformational restrictions that suppress a possible 1,2-hydride shift in the first-formed carbocation and prevent proton transfer to the si face of the C=C π-bond.

#### *epi*-Isozizaene – shape selection

**Figure 11:**
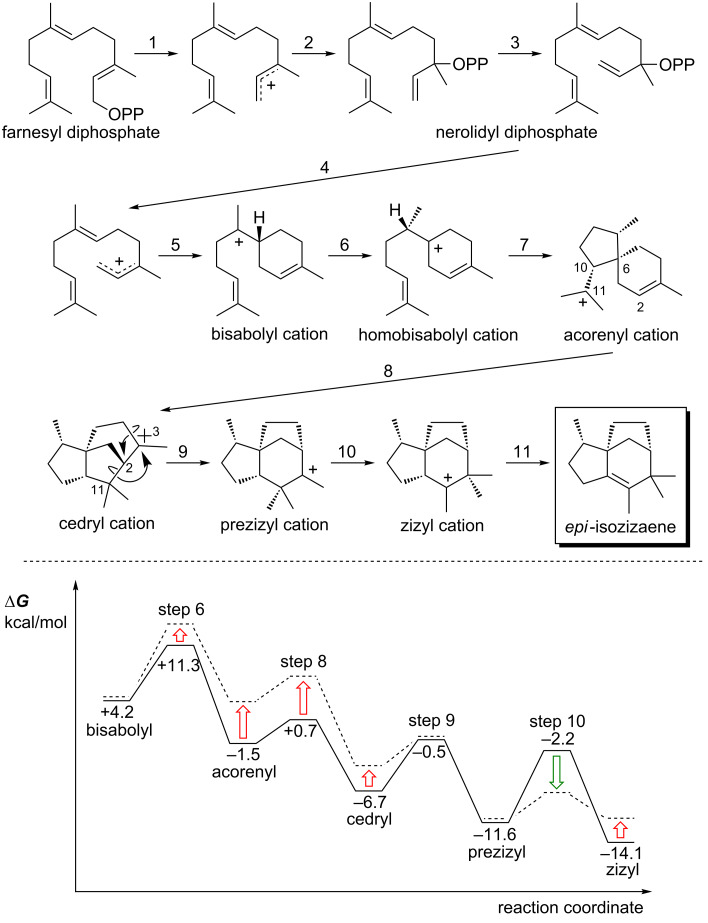
Top: carbocation rearrangements involved in *epi*-isozizaene formation. Bottom: reaction coordinate diagram for conversion of the bisabolyl cation to the zizyl cation in the absence (solid lines; computed relative energies in kcal/mol) and presence (broken lines) of *epi*-isozizaene synthase.

DFT calculations on the pathway for formation of the sesquiterpene *epi*-isozizaene [96–101] (Figure 11) showed that several expected chemical steps were merged into concerted processes [80]. For example, conversion of the homobisabolyl cation to the acorenyl cation (Figure 11, step 7) is barrierless for many conformers of the homobisabolyl cation. In addition, conversion of the cedryl cation to the prezizyl cation involves the combination of two alkyl shift events into a concerted process that avoids formation of a secondary carbocation as a PES minimum (Figure 11, step 9, a “dyotropic” rearrangement) [102]. Direct dynamics trajectory calculations were run for this system starting from the region of the TSS for the 1,2-hydride shift that converts the bisabolyl cation to the homobisabolyl cation (Figure 11, step 6). The goal of this study was to assess how far along the reaction coordinate trajectories would proceed without becoming “trapped” in an intermediate energy well. For some conformers of the bisabolyl cation, many trajectories proceeded to the cedryl cation without significant delay in the regions of the homobisabolyl and acorenyl cations. Subsequent automated docking calculations of carbocations (specifically, those derived from the conformer of the bisabolyl cation that most readily formed the cedryl cation in the dynamics simulations) into the crystallographically-determined structure of *epi*-isozizaene synthase revealed that some carbocations along the reaction coordinate were bound more strongly than others. Of particular note was the prediction that the TSS for conversion of the cedryl cation to the prezizyl cation (Figure 11, step 9) and for the conversion of the prezizyl cation to the zizyl cation (Figure 11, step 10) are bound more strongly than the carbocations that immediately precede them, implying that shape selection by the enzyme can lower the barriers for these steps (Figure 11, bottom), thereby making it more likely that trajectories will proceed to product.

***Take home message:***

• Shape selection by epi-isozizaene synthase can lower barriers for steps in the epi-isozizaene-forming carbocation cascade reaction, thereby increasing the likelihood of direct formation of epi-isozizaene over byproducts.

## Outlook

Clearly, non-statistical dynamic effects play important roles in carbocation rearrangement reactions. Neglecting such dynamic effects may lead to incorrect conclusions about lifetimes of particular structures and product distributions – not merely for reactions of academic interest, but for reactions that occur in Nature during the biosynthesis of complex natural products. While characterizing the dynamical behavior of reactive species is challenging, it can be accomplished using modern computational approaches. We look forward to many more studies that do so. We believe as predicted so presciently by Lionel Salem four and a half decades ago, “…the beautiful mechanistic schemes used by organic chemists to interpret reactions will slowly be supplemented and may eventually be replaced by a detailed picture of the dynamic behavior of the reacting species on a complex potential energy surface” [103].

## References

[1] Christianson D W (2008). Curr Opin Chem Biol.

[2] Christianson D W (2006). Chem Rev.

[3] Davis E M, Croteau R, Leeper F J, Vederas J C (2000). Cyclization Enzymes in the Biosynthesis of Monoterpenes, Sesquiterpenes, and Diterpenes. Biosynthesis.

[4] Cane D E (1999). Compr Nat Prod Chem.

[5] Cane D E (1990). Chem Rev.

[6] Tantillo D J (2011). Nat Prod Rep.

[7] Fukui K (1981). Acc Chem Res.

[8] Maeda S, Harabuchi Y, Ono Y, Taketsugu T, Morokuma K (2015). Int J Quantum Chem.

[9] Eyring H (1935). J Chem Phys.

[10] Fernández-Ramos A, Miller J A, Klippenstein S J, Truhlar D G (2006). Chem Rev.

[11] Truhlar D G, Garrett B C, Klippenstein S J (1996). J Phys Chem.

[12] Laidler K J, King M C (1983). J Phys Chem.

[13] Pechukas P (1981). Annu Rev Phys Chem.

[14] Marcus R A (1952). J Chem Phys.

[15] Marcus R A (1952). J Chem Phys.

[16] Kassel L S (1932). Chem Rev.

[17] Rice O K, Ramsperger H C (1927). J Am Chem Soc.

[18] Pham H V, Houk K N (2014). J Org Chem.

[19] Collins P, Carpenter B K, Ezra G S, Wiggins S (2013). J Chem Phys.

[20] Ess D H, Wheeler S E, Iafe R G, Xu L, Çelebi-Ölçüm N, Houk K N (2008). Angew Chem, Int Ed.

[21] Weitman M, Major D T (2010). J Am Chem Soc.

[22] Major D T, Weitman M (2012). J Am Chem Soc.

[23] Gonzalez C, Schlegel H B (1990). J Phys Chem.

[24] Davies H M L, Lian Y (2012). Acc Chem Res.

[25] Pemberton R P, Hong Y J, Tantillo D J (2013). Pure Appl Chem.

[26] Hong Y J, Tantillo D J (2014). Nat Chem.

[27] Birney D M (2010). Curr Org Chem.

[28] Carpenter B K (1995). J Am Chem Soc.

[29] Lourderaj U, Hase W L (2009). J Phys Chem A.

[30] Carpenter B K (2005). Annu Rev Phys Chem.

[31] Klinman J P (2011). Procedia Chem.

[32] Klinman J P, Kohen A (2013). Annu Rev Biochem.

[33] Klinman J P (2015). Acc Chem Res.

[34] Antoniou D, Caratzoulas S, Kalyanaraman C, Mincer J S, Schwartz S D (2002). Eur J Biochem.

[35] Bachrach S M (2014). Computational organic chemistry.

[36] Lourderaj U, Park K, Hase W L (2008). Int Rev Phys Chem.

[37] Fleming K L, Tiwary P, Pfaendtner J (2016). J Phys Chem A.

[38] Voter A F, Montalenti F, Germann T C (2002). Annu Rev Mater Res.

[39] Xiao P, Duncan J, Zhang L, Henkelman G (2015). J Chem Phys.

[40] Cramer C J (2002). Essentials of computational chemistry: theories and models.

[41] Bogle X S, Singleton D A (2011). J Am Chem Soc.

[42] Saunders M, Telkowski L, Kates M R (1977). J Am Chem Soc.

[43] Saunders M, Kates M R (1977). J Am Chem Soc.

[44] Saunders M, Kates M R, Wiberg K B, Pratt W (1977). J Am Chem Soc.

[45] Ohta B K, Hough R E, Schubert J W (2007). Org Lett.

[46] Ammal S C, Yamataka H, Aida M, Dupuis M (2003). Science.

[47] Roca-López D, Polo V, Tejero T, Merino P (2015). Eur J Org Chem.

[48] Kraka E, Cremer D (2010). Acc Chem Res.

[49] Cremer D, Wu A, Kraka E (2001). Phys Chem Chem Phys.

[50] Duarte F, Gronert S, Kamerlin S C L (2014). J Org Chem.

[51] Joo H, Kraka E, Quapp W, Cremer D (2010). Mol Phys.

[52] Hong Y J, Tantillo D J (2011). Org Lett.

[53] Hong Y J, Tantillo D J (2010). J Am Chem Soc.

[54] Hong Y J, Tantillo D J (2009). J Am Chem Soc.

[55] de Souza M A F, Ventura E, do Monte S A, Riveros J M, Longo R L (2014). Chem – Eur J.

[56] Tantillo D J (2008). J Phys Org Chem.

[57] Tantillo D J (2010). Chem Soc Rev.

[58] Sun L, Song K, Hase W L (2002). Science.

[59] Berson J A, Wege D, Clarke G M, Bergman R G (1969). J Am Chem Soc.

[60] Ghigo G, Maranzana A, Tonachini G (2013). J Org Chem.

[61] Becke A D (1993). J Chem Phys.

[62] Becke A D (1993). J Chem Phys.

[63] Ditchfield R, Hehre W J, Pople J A (1971). J Chem Phys.

[64] Jalife S, Martínez-Guajardo G, Zavala-Oseguera C, Fernández-Herrera M A, von Ragué Schleyer P, Merino G (2014). Eur J Org Chem.

[65] Lobb K A (2015). Eur J Org Chem.

[66] Lawton R G (1961). J Am Chem Soc.

[67] Bartlett P D, Bank S, Crawford R J, Schmid G H (1965). J Am Chem Soc.

[68] Jalife S, Wu J I, Martinez-Guajardo G, von Ragué Schleyer P, Fernandez-Herrera M A, Merino G (2015). Chem Commun.

[69] East A L L, Bucko T, Hafner J (2007). J Phys Chem A.

[70] East A L L, Bučko T, Hafner J (2009). J Chem Phys.

[71] Sandbeck D J S, Markewich D J, East A L L (2016). J Org Chem.

[72] Johnson C K, Collins C J (1974). J Am Chem Soc.

[73] Collins C J, Johnson C K (1973). J Am Chem Soc.

[74] Collins C J, Johnson C K, Raaen V F (1974). J Am Chem Soc.

[75] Tratch S S, Molchanova M S, Zefirov N S (2006). Croat Chem Acta.

[76] Sorensen T S (1976). Acc Chem Res.

[77] Mosley J D, Young J W, Agarwal J, Schaefer H F, von Ragué Schleyer P, Duncan M A (2014). Angew Chem, Int Ed.

[78] Hong Y J, Tantillo D J (2010). Org Biomol Chem.

[79] Lodewyk M W, Gutta P, Tantillo D J (2008). J Org Chem.

[80] Pemberton R P, Ho K C, Tantillo D J (2015). Chem Sci.

[81] Hong Y J, Tantillo D J (2013). Chem Sci.

[82] Lafever R E, Vogel B S, Croteau R (1994). Arch Biochem Biophys.

[83] Peters R J, Flory J E, Jetter R, Ravn M M, Lee H-J, Coates R M, Croteau R B (2000). Biochemistry.

[84] Peters R J, Croteau R B (2002). Proc Natl Acad Sci U S A.

[85] Peters R J, Ravn M M, Coates R M, Croteau R B (2001). J Am Chem Soc.

[86] Funk C, Croteau R (1994). Arch Biochem Biophys.

[87] Ravn M M, Peters R J, Coates R M, Croteau R (2002). J Am Chem Soc.

[88] Ravn M M, Coates R M, Jetter R, Croteau R B (1998). Chem Commun.

[89] Ravn M M, Coates R M, Flory J E, Peters R J, Croteau R (2000). Org Lett.

[90] Wilderman P R, Peters R J (2007). J Am Chem Soc.

[91] Hong Y J, Tantillo D J (2009). Nat Chem.

[92] Siebert M R, Zhang J, Addepalli S V, Tantillo D J, Hase W L (2011). J Am Chem Soc.

[93] Siebert M R, Manikandan P, Sun R, Tantillo D J, Hase W L (2012). J Chem Theory Comput.

[94] Gao W, Hillwig M L, Huang L, Cui G, Wang X, Kong J, Yang B, Peters R J (2009). Org Lett.

[95] Hong Y J, Tantillo D J (2015). J Am Chem Soc.

[96] Aaron J A, Lin X, Cane D E, Christianson D W (2010). Biochemistry.

[97] Lin X, Hopson R, Cane D E (2006). J Am Chem Soc.

[98] Zhao B, Lin X, Lei L, Lamb D C, Kelly S L, Waterman M R, Cane D E (2008). J Biol Chem.

[99] Lin X, Cane D E (2009). J Am Chem Soc.

[100] Li R, Chou W K W, Himmelberger J A, Litwin K M, Harris G G, Cane D E, Christianson D W (2014). Biochemistry.

[101] Belhassen E, Baldovini N, Brevard H, Meierhenrich U J, Filippi J-J (2014). Chem Biodiversity.

[102] Gutierrez O, Tantillo D J (2012). J Org Chem.

[103] Salem L (1971). Acc Chem Res.

