# Correction: Dynamic behavior of rearranging carbocations – implications for terpene biosynthesis

**DOI:** 10.3762/bjoc.13.161

**Published:** 2017-08-15

**Authors:** Stephanie R Hare, Dean J Tantillo

**Affiliations:** 1 Department of Chemistry, University of California–Davis, 1 Shields Avenue, Davis, CA 95616, USA

**Keywords:** carbocation, density functional theory, dynamics, mechanism, terpene

The originally published Figure 6 had several mis-drawn structures with charges in incorrect locations. These errors have been corrected in the new version (see Figure 1).

**Figure 1:**
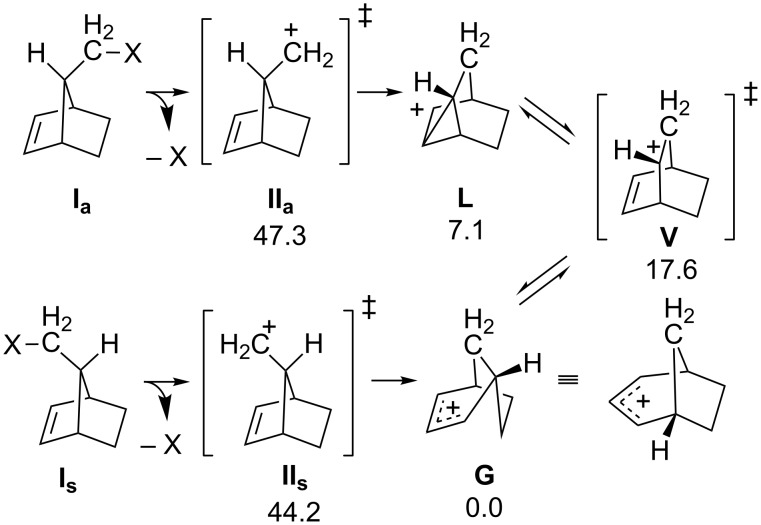
Corrected Figure 6 of the original article. The portion of the norborn-2-en-7-ylmethyl cation PES examined by Ghigo et al. [60]. Energies reported are electronic energies, including zero-point corrections (ZPE), at the B3LYP/6-31G(d) level of theory and are all relative to that of **G** [61-63] (for references see original article).

